# Dietary differences in archosaur and lepidosaur reptiles revealed by dental microwear textural analysis

**DOI:** 10.1038/s41598-019-48154-9

**Published:** 2019-08-12

**Authors:** Jordan Bestwick, David M. Unwin, Mark A. Purnell

**Affiliations:** 1 School of Geography, Geology and the Environment, University of Leicester, Leicester, LE1 7RH United Kingdom; 2 School of Museum Studies, University of Leicester, Leicester, LE1 7RF United Kingdom

**Keywords:** Evolutionary ecology, Evolution

## Abstract

Reptiles are key components of modern ecosystems, yet for many species detailed characterisations of their diets are lacking. Data currently used in dietary reconstructions are limited either to the last few meals or to proxy records of average diet over temporal scales of months to years, providing only coarse indications of trophic level(s). Proxies that record information over weeks to months would allow more accurate reconstructions of reptile diets and better predictions of how ecosystems might respond to global change drivers. Here, we apply dental microwear textural analysis (DMTA) to dietary guilds encompassing both archosaurian and lepidosaurian reptiles, demonstrating its value as a tool for characterising diets over temporal scales of weeks to months. DMTA, involving analysis of the three-dimensional, sub-micrometre scale textures created on tooth surfaces by interactions with food, reveals that the teeth of reptiles with diets dominated by invertebrates, particularly invertebrates with hard exoskeletons (e.g. beetles and snails), exhibit rougher microwear textures than reptiles with vertebrate-dominated diets. Teeth of fish-feeding reptiles exhibit the smoothest textures of all guilds. These results demonstrate the efficacy of DMTA as a dietary proxy in taxa from across the phylogenetic range of extant reptiles. This method is applicable to extant taxa (living or museum specimens) and extinct reptiles, providing new insights into past, present and future ecosystems.

## Introduction

Extant reptiles include lepidosaurs (lizards, snakes and tuataras), archosaurs (crocodilians) and chelonians (turtles and tortoises). They live in terrestrial, semi-aquatic and marine ecosystems around the world^1^, and perform key roles in ecosystem functioning, acting as apex predators^2,3^, seed-dispersers^4,5^ and opportunists^6^. One-fifth of all reptile species are currently threatened with extinction by factors such as climate change and habitat fragmentation^7^, and detailed characterisations of their ecological roles, including trophic interactions, are urgently required if effective population and habitat management strategies are to be implemented^8^. Our understanding of reptile diets at the species level, however, is patchy^9–11^.

Several proxies are currently used to reconstruct reptile diets, but they are rarely used in concert and often record information over different temporal scales. Feeding observations, for example, only provide information when animals can be directly observed, which can quickly become time and labour intensive, especially if subjects feed nocturnally, underwater or within tree canopies^12,13^. Stomach content analysis, identification of the frequency and/or volume of food items within an individual’s stomach, generally provides evidence of items consumed within the previous few hours or days^13^. Acquiring representative sample sizes can present practical problems (e.g. ref.^14^), and this type of data may also be biased by indigestible items and by secondary ingestion^15,16^. Stable isotope analysis of soft tissues such as blood and muscles, and of hard tissues such as teeth, claws and scales is widely used, but captures dietary information averaged over months to years^17–19^, and yields only coarse indications of relative trophic levels occupied^10,20,21^. Analysis of reptile tooth and jaw morphology can provide dietary information over evolutionary time-scales^11^, and has been widely used for reconstructing the diets of extinct reptiles^12^. However, this type of analysis generally provides only coarse indications of trophic interactions and assumes close relationships between morphology and hypothesised functions of structures^22,23^, which in many cases has demonstrably been shown to be false^24^. Hence, there is a need for techniques that: (i) provide dietary information over temporal scales that are longer than those of feeding observations and stomach content analyses, but shorter and more specific than those of stable isotope analyses; (ii) allow identification of population variation within a species and; (iii) are not underpinned by assumptions of a close relationship between morphology and function^18^.

Dental Microwear Textural Analysis (DMTA) offers a potential solution. DMTA involves quantitative analysis of the sub-micrometre scale three-dimensional textures that form as wear patterns on tooth surfaces during food consumption^22,25–28^. Microwear formation is determined by the relative difficulty experienced by consumers in piercing and chewing food items (i.e. the ‘intractability’ of foodstuffs^29^, here referred to for simplicity as ‘hardness’ – see methods), providing a direct record of the nature of food consumed^30–33^. Analyses of three-dimensional tooth surface textures use standardized texture parameters^25,26,34,35^ to quantify microwear characteristics and identify dietary differences between populations and/or species, an approach that is effective even with limited sample sizes^33,36,37^. DMTA studies have primarily focused on the occlusal facets (chewing surfaces) of mammalian teeth (see ref.^38^ for a review), with the microwear dietary signal recording items consumed from the previous few weeks^39–41^. This has allowed identification of seasonal dietary differences in mammals^40,42^, and combined with multivariate analysis provides a robust framework for dietary reconstructions of extinct taxa (e.g. ref.^43^). DMTA is potentially applicable to reptiles, but they differ in a number of significant ways from mammals: (i) their teeth are routinely shed^44^; (ii) dentitions are typically non-occlusal; (iii) food processing is less (reptiles commonly swallow entire food items or crudely tearing them into pieces^45,46^); (iv) they have lower energy requirements because of their ectothermic metabolism^47^ and thus consume less. These characteristics limit the frequency and duration of tooth-food interactions in reptiles, relative to those of mammals, and differences in microwear textures between reptiles with different diets are likely to be more subtle and to reflect diet over longer temporal scales.

Recently, an analysis of lepidosaurs found evidence of differences in dental microwear texture between specialised dietary groups (e.g. between carnivorous and frugivorous species)^48^. However, because the analysis was limited to lepidosaurs, the hypothesis that microwear texture records dietary signals in other reptile groups, including archosaurs (which includes dinosaurs), remains untested. The anatomy and functional morphology of archosaur and lepidosaur jaws differ in several ways. For example, archosaur teeth are implanted within sockets in the jaw, while lepidosaur teeth are implanted either to the sides or apices of the jaw^49,50^. In addition, modern crocodilians have some of the highest bite forces among modern taxa^51^, while lepidosaurs have comparatively weaker bites^52^. It remains unknown whether these anatomical or functional differences influence microwear formation and obscure dietary signals between unrelated reptiles. Until the hypothesis that microwear tracks diet has been tested, DMTA cannot be considered as a reliable proxy for dietary reconstruction in non-lepidosaur reptiles.

**Figure 1:**
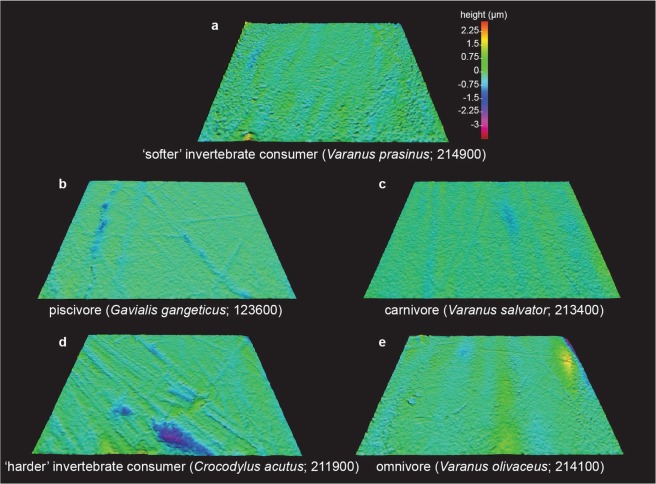
Examples of scale-limited reptile tooth surfaces for each dietary guild. (**a**) ‘Softer’ invertebrate consumer (*Varanus prasinus*). (**b**) Piscivore (*Gavialis gangeticus*). (**c)** Carnivore (*Varanus salvator*). (**d)** ‘Harder’ invertebrate consumer (*Crocodylus acutus*). (**e)** Omnivore (*Varanus olivaceus*). Measured areas are 146 × 110 µm in size. Topographic scale in micrometres. Numbers within brackets denote Leicester IFM sample numbers.

Here, we present the first application of DMTA to reptile dietary guilds that encompasses both archosaur and lepidosaur species. Data from crocodilians (Crocodylia) and varanid lizards (Varanidae) with well-constrained dietary differences (based on stomach content and/or stable isotope analyses; see Supplementary Table S1) was used to test the null hypotheses that microwear textures do not differ between reptile dietary guilds, and to explore how textures differ between guilds. We sampled six crocodilian and seven varanid species, each independently assigned to one of five dietary guilds: carnivores (*Crocodylus porosus* ‘adults’, *Varanus komodoensis*, *Varanus nebulosus*, *Varanus rudicollis*, *Varanus salvator*), consumers of ‘harder’ invertebrates (*Crocodylus acutus*, *Crocodylus porosus* ‘juveniles’), consumers of ‘softer’ invertebrates (*Varanus niloticus*, *Varanus prasinus*), omnivores (*Varanus olivaceus*) and piscivores (*Alligator mississippiensis*, *Caiman crocodilus*, *Crocodylus niloticus*, *Gavialis gangeticus*; see Methods and Supplementary Fig. S1 for dietary guild descriptions and Fig. 1 for examples of digital elevation models of scale-limited tooth surfaces from which texture data were acquired.

## Results

The results of the ANOVA and additional tests allow us to reject the null hypothesis. Four tooth surface texture parameters differ significantly between reptiles from different dietary guilds: Spk, Sds, Vmp, and; Smr1 (Tables 1 and S2, and Supplementary Fig. S2 provide explanations of the ISO parameters^35^, see Table 2 and the Supplementary Text for ANOVA and Tukey HSD pairwise results).

**Table 1:** Definition, description, and categorization of the International Organisation for Standardisation (ISO) 3D texture parameters that correlate with PC axes.

Parameter	Unit	Def inition		
Sq	µm	Root-Mean-Square height of surface	height	Sq, root mean square of height, provides an overall measure of the height of the texture comprising the surface
Sp	µm	Maximum peak height of surface	height	Sp, the height of the highest peak, tends not to be reliable as an indicator of overall surface texture, as it is based on only one peak, which could reflect a single point.
Sz	µm	Maximum height of surface	height	Sz, the maximum height of the surface, is calculated by subtracting the maximum valley depth from the peak height
Sa	µm	Average height of surface	height	Sa, the average height of the surface, provides an overall measure of the height of the texture comprising the surface
S5z	µm	10 point height of surface	feature	S5z is the average value of the 5 highest peaks and the 5 deepest valleys, providing a measure of extremes of height
Sds	1/mm^2^	Density of summits. Number of summits per unit area making up the surface	hybrid	Increases in Sds indicate that peaks above the core material make up a greater proportion of the surface
Ssc	1/µm	Mean summit curvature for peak structures		Increases in Ssc indicate that peaks above the core material have more rounded summits
Sk	µm	Core roughness depth, Height of the core material	material ratio	The vertical distance between the low and high limits of the core material (defined as outlined in table caption and Fig. S2)
Spk	µm	Mean height of the peaks above the core material	material ratio	Spk is the mean height of peaks above the top of the core, with high values indicating a surface composed of high peaks
Svk	µm	Mean depth of the valleys below the core material	material ratio	Spk is the mean depth of valleys below the base of the core, with high values indicating a surface composed of deep valleys
Smr1	%	Surface bearing area ratio (the proportion of the surface which consists of peaks above the core material)	material ratio	The percentage of the surface that is composed of the peaks that are higher than the top of the core.
Smr2	%	Surface bearing area ratio (the proportion of the surface which would carry the load)	material ratio	The percentage of the surface that is composed of the valleys that are lower than the base of the core.
Vmp	µm^3^/mm^2^	Material volume of the peaks of the surface	volume	The volume of material contained within peaks that make up the highest 10% of the surface
Vmc	µm^3^/mm^2^	Material volume of the core of the surface	volume	The volume of the material making up the surface, excluding peaks (the highest 10%) and valleys (lowest 20% of the surface). ‘Core’ in the context of volume parameters is not defined in the same way as core for material ratio parameters.
Vvc	µm^3^/mm^2^	Void volume of the core of the surface	volume	The volume of the voids within the ‘core’ of the surface, the core excluding peaks (the highest 10%) and valleys (lowest 20% of the surface).
Vvv	µm^3^/mm^2^	Void volume of the valleys of the surface	volume	The volume of voids contained within valleys that make up the lowest 20% of the surface.

Many parameters are derived from the areal material ratio curve; a cumulative probability density function derived from the scale-limited tooth surface by plotting the cumulative percentage of the tooth surface against height. Fig. S2 provides a graphical explanation. The peaks, valleys and core material of tooth surfaces are defined on the basis of this curve: parts of the surface that are higher or lower than the core are defined as peaks and valleys respectively. A full listing of parameter definitions is provided in Table S2.

**Table 2:** ANOVA results (4 d.p) of ISO texture parameters between reptile dietary guilds.

Parameter	*F*-ratio	*P*-value	d.f
Sq	2.5649	0.0435	4, 90
Sku	1.678	0.162	4, 90
Sp	2.2435	0.0705	4, 90
Sv	1.345	0.2595	4, 90
Sz	1.7548	0.145	4, 90
**Sds**	**5**.**2453**	**0**.**0008**	**4**, **90**
Str	1.9465	0.1096	4, 90
Sdq	1.1949	0.3186	4, 90
Ssc	2.6308	0.0394	4, 90
Sdr	1.2601	0.2916	4, 90
**Vmp**	**4**.**1834**	**0**.**0037**	**4**, **90**
Vmc	1.5956	0.1823	4, 90
Vvc	2.3298	0.062	4, 90
Vvv	1.2956	0.2778	4, 90
**Spk**	**4**.**0231**	**0**.**0048**	**4**, **90**
Sk	1.8817	0.1205	4, 90
Svk	1.6775	0.1621	4, 90
**Smr1**	**4**.**2863**	**0**.**0032**	**4**, **90**
Smr2	1.6954	0.158	4, 90
S5z	1.0669	0.3776	4, 90
Sa	2.0288	0.097	4, 90

Data log transformed and scale limited using 5^th^ order polynomial and robust Gaussian filter. Texture parameters exhibiting significant differences after application of the Benjamini-Hochberg procedure shown in bold.

**Figure 2:**
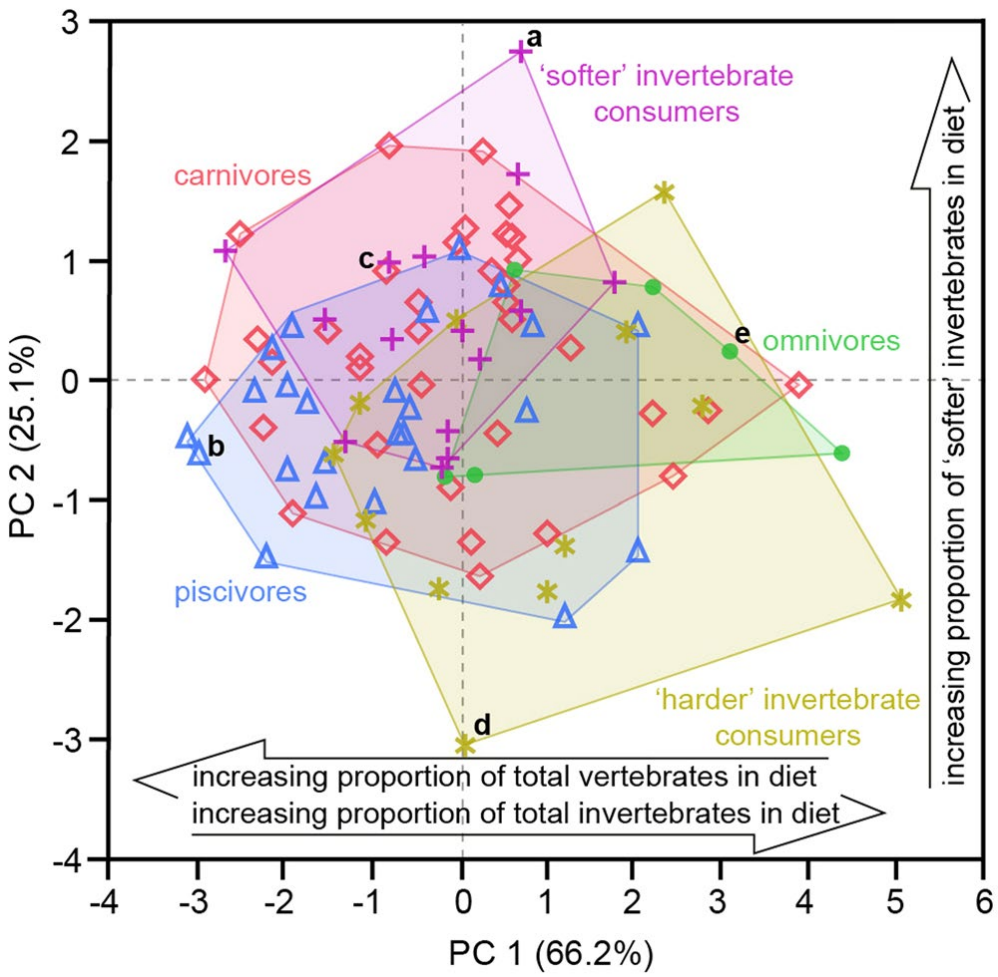
Reptile dietary discrimination. Principal Component Analysis (PCA) of the four ISO texture parameters (Spk, Sds, Vmp and Smr1) that distinguish reptile dietary guilds. (**a**–**e**) indicate the examples of scale limited textures illustrated in Fig. 1. Arrows show significant correlations of dietary characteristics along PC axes 1 and 2. For full parameter definitions and details of all dietary correlations along PC axes 1 and 2, see the Supplementary Information.

Principal Component Analysis (PCA) of these four parameters separates reptile guilds in a multivariate texture-dietary space, defined by principal component axes 1 and 2, which together account for 91.3% of the total variance (Fig. 2). Increasingly negative values along PC axis 1 (66% of variance) correlate with higher proportions of total vertebrates in reptile diets (*r*_*s*_ = −0.3564, *P* = 0.0004), while increasingly positive values correlate with higher proportions of total invertebrates (*r*_*s*_ = 0.3192, *P* = 0.0016). Increasingly positive values along PC axis 2 correlate with higher proportions of ‘softer’ invertebrates (*r*_*s*_ = 0.2907, *P* = 0.0043; for all dietary correlations along PCs 1 and 2, see Supplementary Table S3). ANOVA of the PCA results provides evidence of additional discriminatory power: PC axes 1 and 2 differ between dietary guilds (PC 1, *F* = 4.9316, d.f. = 4, 90, *P* = 0.0012; PC 2, *F* = 4.6676, d.f. = 4, 90, *P* = 0.0018); piscivores differ from ‘harder’ invertebrate consumers and omnivores (PC axis 1, Tukey HSD); ‘harder’ invertebrate consumers differ from carnivores and ‘softer’ invertebrate consumers; and ‘softer’ invertebrate consumers differ from piscivores (PC axis 2, Tukey HSD).

The PCA allows exploration of the relationship between microwear textural differences and the texture-dietary space. Seventeen texture parameters correlate positively with PC axis 1 (Supplementary Table S4); in very broad terms, surfaces get rougher as diet changes. This is a gross simplification, however, and does not capture how significant aspects of the surface texture change. This requires us to consider the nature of the texture parameters, and for clarity we focus on the 12 that exhibit the strongest correlations (*r*_*s*_ > 0.5). Sa, Sq, Sp, Sz, S5z capture aspects of the height of surfaces, and together they indicate that as diet changes along PC 1, the height of the texture surface increases (see Table 1 for more details; Sp and Sz are derived from the height of a single point in a surface and are therefore not reliable as indicators of the overall surface and are not considered further). The remaining seven parameters are defined relative to the ‘core’ of the material making up the surface (Table 1). Together, the parameters correlated with PC 1 indicate that as diet changes along PC 1 (differences in carnivory, piscivory and consumption of invertebrates) surfaces are higher (Sa, Sq, S5z), increasingly composed of high peaks (Spk, Smr1, S5z) with increasing peak volume (Vmp); the core increases in depth (Sk), and as it does so contains more material and void volume (Vmc, Vvc). Table 1 provides details of the aspects of surface texture that these parameters capture.

Nine parameters exhibit correlations with PC axis 2, but only two correlate strongly (*r*_*s*_ > 0.5; Supplementary Table S4). Sds and Ssc both increase, indicating that as diet changes, and the relative quantity of ‘softer’ invertebrates consumed increases, peaks above the core material make up a greater proportion of the surface, and the peaks themselves become more rounded. Of the remaining seven parameters, five exhibit significant but weaker correlations with PC 2, at a similar level to one another (*r*_*s*_ = 0.3 ± 0.02). As diet changes, Svk and Smr2 increase and Vvv decreases, indicating that the deeper valleys in the surface decrease in mean depth (Svk) and total void volume (Vvv) but make up an increasing proportion of the surface (Smr2). Sdq and Sdr both increase, indicating steeper gradients of the slopes comprising the surface, and more intricate, complex textures.

Further exploratory analysis reveals more textural differences associated with diet. Matched Pairs t-tests comparing the average parameter values for guilds (Supplementary Table S5) reveal that piscivores differ significantly from all other guilds – having the lowest average values (4^th^ or 5^th^ rank; Supplementary Table S6) for 17 of 21 parameters, including 16 of the 17 parameters that correlate with dietary axis PC 1 – and carnivores differ significantly from ‘harder’ invertebrate consumers. Compared to other guilds, piscivore teeth, in a general sense, are smoother (Fig. 1), having microwear textures with lower surfaces (Sa, Sq, S5z) and fewer high peaks (Spk, Smr1, S5z) with less peak volume (Vmp) and cores that are reduced in depth (Sk) and contain less material and void volume (Vmc, Vvc).

Omnivore textures exhibit the highest average values for nine of the 17 parameters that correlate with dietary axis PC 1. Compared to other guilds, their surface textures are higher (Sq, Sz) with higher peaks (Sp) and they are spikey (high Sku values), with more high peaks (Spk, Smr1) comprising a greater peak volume (Vmp). They have greater maximum valley depths (Sv), with valleys making up a higher proportion of the surface texture (Smr2) (Fig. 1). Similarly, ‘harder’ invertebrate consumers exhibit high average values for parameters correlated with diet, these being highest for five of the 17 parameters that correlate with PC 1 (Vmc, Vvc, Vvv, Sk, S5z and Sa), and highest for the two parameters that correlate with PC 1 (Vmc, Vvc, Vvv, Sk, S5z, Sa), and highest for the two parameters that correlate negatively with PC 2 (Svk and Vvv). Compared to other guilds, their surface textures are higher (Sa, S5z) and have a deeper core (Sk) with greater void volume (Vvv). Putting this simply, teeth of omnivores and ‘harder’ invertebrate consumers have rougher surface textures (Fig. 1).

## Discussion

This study provides the first evidence that microwear textures on non-occlusal tooth surfaces in reptile dietary guilds that encompass archosaurs and lepidosaurs track dietary differences and differ between guilds. In particular, differences in microwear texture correlate with dietary differences in proportions of total vertebrates, total invertebrates and ‘softer’ invertebrates. Reptiles that consume greater proportions of ‘harder’ foods have microwear textures that, in general terms, are rougher. This is broadly similar to the only previous study of reptile microwear texture^48^, which found that molluscivorous reptiles had the highest height and volume ISO parameters (Sa, Sq, Sdq, Vvv, Vmc and Vvv) for all guilds surveyed. The results of the present study are also consistent with previous ISO-based analyses of non-occlusal tooth surfaces for other groups of vertebrates. For example, of the 12 parameters that exhibit strong correlations with diet in reptiles (PC1) all but two were found in cichlid fishes to correlate with a qualitative ranking of microwear roughness in cichlid fishes. Several height and volume parameters (Sa, Spk, Sk, Vmp, Vmc, and Vvc) differ significantly between the non-occlusal, labial surfaces of oral teeth in cichlids feeding from substrates that differed in their abrasiveness^22^. We are aware of only three other studies of microwear texture and diet on non-occlusal tooth surfaces, but none were based on ISO parameters, so direct comparison of texture attributes with our study is not possible. Nonetheless, these studies found significant relationships between diet/feeding and microwear textures on the labial surfaces of shrew incisors^53^, the labial surfaces of incisors in platyrrhine primates (New World primates)^54^ and the buccal surfaces of molars in catarrhine primates (Old World primates)^55^.

Although less relevant, ISO-based analyses of *occlusal* surfaces have reported similar patterns to those found in our study. In bats, Sa, Sq, Sk, Svk, Vmp, Vmc, and Vvc differ between species and correlate positively with differences in the proportions of ‘hard’ food in their diets^33^. Other studies have also found comparable height and volume parameters to differ between molars of primates and pig species with different diets^56,57^, but the relationship with the properties of the consumed food is less clear.

In the absence of detailed experimental studies, the nature of the potential causal relationships between dental microwear texture and diet in reptiles is a matter of speculation, but the correlations between texture parameters and diets that clearly differ in terms of the properties of the food consumed is certainly suggestive. Unlike the majority of microwear studies which focus on occlusal molar facets with texture developed through the compressive and shearing forces of chewing and tooth-food-tooth interactions, the texture developed on the non-occlusal surfaces analysed in this study must primarily reflect tooth-food interactions resulting from prehension, biting and piercing. This is also a likely contributor, in part, to the greater degree of overlap between dietary guilds seen in reptile microwear, compared to previous studies of mammal molar microwear texture. This is to be expected because chewing in mammals, with repeated compression and shearing of food between two opposed enamel surfaces, increases the frequency of tooth-food interactions, and thus the frequency with which microwear features are generated. Compared to the mesial teeth sampled for our study, the forces operating to generate microwear features, patterns and textures will also be greater in mammal molars. Simply put, occlusal facets in mammal molars are more predisposed to the development of a close relationship between microwear texture and the properties of the food consumed than are the non-occclusal surfaces of reptile teeth. The lower frequency of interactions with food (lack of true chewing) and the lower forces acting on non-occlusal tooth surfaces leads to the expectation that microwear traces will be less distinctive. This is supported by analysis of non-occlusal microwear texture in bats, which found significant differences combined with overlap between guilds (Bestwick *et al*. *unpublished data*). An additional potential explanation is that reptiles exhibit greater inter-individual variation in diet. This can be quite marked^6,58^ and has recently been cited as a possible cause of overlap in microwear texture parameter values between dietary guilds in lepidosaurs^48^. Nonetheless, despite their differences from mammals, and the expectation that this would weaken the relationship between microwear texture and diet, we find significant and informative differences between reptiles from different dietary guilds.

The teeth of reptiles that generally consume the highest proportions of invertebrates, in this case arthropods and shelled gastropods, exhibit the roughest microwear textures with height and volume parameters generating the highest values. The chitinous exoskeletons of arthropods and the shells of gastropods are relatively hard, not only requiring fracture before these organisms can be effectively digested^29^, but are also more likely to have an impact on the generation of microwear texture. Our results are consistent with experimental work which shows that higher bite forces are required to fracture these structures^59–61^. Likewise, the lower height and volume parameter values generated by a diet of ‘softer’ invertebrates are not unexpected, given that ‘softer’ invertebrates do not require as much force to fracture^59–62^.

Consideration of the material properties of food also explains the similarity of rough microwear textures in omnivores and consumers of ‘harder’ invertebrates. The omnivore from which we obtained our data, *Varanus olivaceus*, orally processes the ‘hard’, woody endocarps that encase dietary fruits^63,64^, and is also known to include snails in its diet as do ‘harder’ invertebrate consumers^17,63^. Interestingly, although our DMTA results are consistent with feeding observations and stomach content analyses, the isotopic composition of *V*. *olivaceus* claws is more similar to that of carnivorous varanids than to insectivorous varanids or unrelated herbivorous lizards^17^. Presumably, tetrapods form a significant part of the diet of *V*. *olivaceus* over longer temporal scales. This highlights the need for evidence across a range of temporal scales to build comprehensive models of diet in reptiles^12^.

Consumers of ‘softer’ invertebrates and carnivores provide another example of similarities in dental microwear textures (although carnivores exhibit some differences, such as slightly higher values for height and volume parameters). The DMTA results reflect a degree of dietary overlap between these guilds, evidenced by stomach content analysis and observational studies. Some carnivores in our study, including *V*. *rudicollis* and *V*. *salvator*, are rather generalistic and consume some ‘softer’ invertebrates, while some of the ‘softer’ invertebrate consumers include mammals in their diet (Supplementary Table S1). DMTA thus successfully reflects the dietary overlap between these reptiles over temporal scales of weeks to months.

Importantly, DMTA effectively discriminates between guilds that exhibit little to no dietary overlap. For example, carnivore tooth microwear textures exhibit lower values for height, volume and material ratio parameters compared to ‘harder’ invertebrate consumers. The carnivores studied primarily consume mammals, amphibians and birds, all of which have unmineralised external integuments^65^. Although the ‘hardness’ of these foods is poorly understood, several carnivores including adult *Cr*. *porosus* and *V*. *komodoensis* are renowned apex predators^2,66^ and rarely consume invertebrates. Furthermore, individual tetrapods have a higher calorific value than individual invertebrates^47^, thus fewer food items need to be consumed to meet metabolic requirements, presumably resulting in fewer tooth-food interactions (although large tetrapods require more oral handling^45,46^). These factors may explain why the teeth of reptilian carnivores generally lack textures that are associated with consumption of ‘harder’ items^22,23,41,56,67,68^.

That piscivore teeth exhibit the smoothest microwear textures of any guild is somewhat surprising. This is unlikely to represent an aquatic versus terrestrial feeding signal since ‘harder’ invertebrate consuming crocodilians also feed in water^69,70^ as does the carnivore *V*. *salvator*^68^, although to a lesser extent. Generally, the piscivores studied consume very few invertebrates (Supplementary Table S1), which may explain textural differences compared to predominantly invertebrate-feeding guilds^22,56^. Why tooth textures differ between piscivores and carnivores, however, is less clear. The ‘hardness’ of fish scales is poorly known, but piscivore microwear textures might have been expected to have been rougher than carnivore textures, the latter primarily feeding on tetrapods that lack structurally reinforced or mineralised integuments^65^. While the impact of invertebrate consumption by some carnivores cannot be ruled out, textural differences between piscivores and carnivores may be attributable to food processing behaviours. The teeth of some carnivores, for example, acquire surface scoring from bones when tearing pieces of flesh from carcasses^45,46^, whereas piscivores almost invariably swallow fish whole after capture^45^. Comparable textural differences are present in mammalian carnivores that consume only the softer parts of carcasses, i.e. muscles and skin: they have smoother microwear textures than taxa whose teeth interact with bones^71^. That similar trends in microwear exist in such distantly related taxa provides further evidence in support of the use of DMTA for reconstructing reptile diets.

In conclusion, this study demonstrates that dental microwear textures on non-occlusal tooth surfaces differ between dietary guilds that encompass archosaurs and lepidosaurs. Microwear textures of invertebrate consumers and omnivores are, in general terms, rougher than carnivore textures, which in turn are rougher than piscivore textures. Although there have been few studies on non-occlusal microwear, these differences match the relationships between diet and microwear reported from lepidosaurian reptiles, mammals and fishes^22,33,41,48,53,55,56,68,71^. DMTA thus offers an independent technique for reconstructing reptile diets over weeks to months, complementing proxies derived from analysis of stomach contents and stable isotopes in reconstructing diets across multiple temporal scales. Such multi-proxy approaches have the potential to increase our understanding of how reptiles utilise food resources and allow better characterisation of competition and niche partitioning within ecosystems^12,13,72^. This will enable better informed predictions of how reptile diets and their ecosystems may change in response to global change drivers^8,72^.

A further advantage of DMTA is that it does not require assumptions of close relationships between tooth form and function^22,23^, assumptions that can hamper morphology-based dietary reconstructions of past ecosystems and trophic interactions. Microwear data can be acquired from historic museum specimens to reconstruct diets from reptile populations living under different environmental conditions, providing baseline data against which to identify dietary shifts driven by anthropogenic environmental change, and inform predictions of how ecosystems will respond. Moreover, our analysis indicates that DMTA tracks diet across a wide phylogenetic range, with dietary signals preserved in reptile guilds that include derived archosaurs and lepidosaurs. This, in turn, indicates that DMTA of extant reptiles can provide a robust multivariate framework within which to test hypotheses and constrain the diets of extinct reptiles with non-occlusal dentitions (e.g. dinosaurs, pterosaurs, ichthyosaurs and plesiosaurs), providing new insights into extinct ecosystems^28^.

## Methods

### Study species and material sampled

We sampled tooth microwear textures for six crocodilian species and seven varanid species from specimens in: the Field Museum of Natural History, Chicago, Illinois, USA (FMNH); Grant Museum of Zoology, University College London, London, UK (LDUCZ); Natural History Museum, London, UK (NHMUK); University of Oxford Museum of Natural History, Oxford, UK (OUMNH); Florida Museum of Natural History, Gainesville, Florida, USA (UF); and the National Museum of Natural History, Smithsonian Institute, Washington D.C., USA (USNM) (Supplementary Table S7). Where possible, specimens of the same ontogenetic stage, usually adults, were sampled to minimise noise introduced from unknown ontogenetic dietary differences and more rapid tooth shedding rates in younger individuals^44^. Captive raised specimens were not sampled as their diets are likely to have differed from dietary data reported for wild specimens.

### Dietary guild assignments

Previous ecological studies have assigned animals to dietary guilds according to the food group that makes up more than ≥50% of their diet by volume or frequency^6,73–75^. Alternatively, they have simply classified animals according to the proportions of animal and/or plant matter consumed^9,11,76^. We have adapted these classifications by taking into account the relative ‘intractability’ of prey as food^29^, but the term is not widely used so we utilise ‘hard’ and ‘soft’ to mean prey that is more or less difficult to pierce and process. Reptiles that primarily consume vertebrates were assigned to one of two guilds according to whether they mainly eat tetrapods or fish. Reptiles that primarily consume invertebrates were assigned to one of three guilds according to the relative intractability of invertebrates consumed. Invertebrates were assigned to one of three classes based on the relative hardness of their exoskeletons, assessed according to experimental work aimed at quantifying this value^59–62^. ‘Harder’ invertebrates include those with hard exoskeletons: coleopterans, crustaceans and shelled gastropods; ‘softer’ invertebrates include those with less hard exoskeletons, such as orthopterans, formicid hymenopterans and odonatans, which are in turn harder than the ‘softest’ invertebrates, such as invertebrate larvae, lepidopterans, araneans and myriapods.

Information on diets was compiled from stomach and/or faecal content analyses^2,6,58,63,68,77–84^ (Supplementary Table S1). Where possible, these were selected to meet all the following criteria: representative sample sizes; dietary compositions presented as volumetric data; and spatial proximity of the content studies to the location(s) from which the museum specimens we analysed were collected.

The availability of museum specimens allowed multiple life-history stages of *Crocodylus porosus* to be included as this species exhibits ontogenetic niche partitioning^69,81^. Specimens with a total length of less than 3.5 m were classified as juveniles; specimens with total lengths exceeding 3.5 m were considered to be adults^85^.

*Crocodylus porosus* ‘adults’, and individuals of *Varanus komodoensis*, *Varanus nebulosus*, *Varanus rudicollis* and *Varanus salvator* were assigned to the carnivore guild (tetrapod consumers, *n* = 37); individuals of *Crocodylus acutus*, and *Crocodylus porosus* ‘juveniles’ were assigned to the ‘harder’ invertebrate consumer guild (*n* = 12); individuals of *Varanus niloticus* and *Varanus prasinus* were assigned to the ‘softer’ invertebrate consumer guild (*n* = 15); individuals of *Varanus olivaceus* were assigned to the omnivore guild (*n* = 6); individuals of *Alligator mississippiensis*, *Caiman crocodilus*, *Crocodylus niloticus* and *Gavialis gangeticus* were assigned to the piscivore guild (*n* = 25). For a schematic overview of how reptiles were classified, see Supplementary Fig. S1.

*Ca*. *crocodilus* exhibits seasonal dietary shifts, consuming more fish in the wet season (March–May) and more invertebrates in the dry season (August–September)^78^. DMTA can identify seasonal dietary shifts in mammals^39,40,42,86^, and the same may be true of reptiles. Consequently, sampled *Ca*. *crocodilus* specimens that had died early in the dry season were classified as piscivores under the assumption that their tooth surface textures retained the piscivore signal accumulated during the wet season. The unavailability of specimens prevented the sampling of *Ca*. *crocodilus* individuals that had died during the wet season.

### Sampling strategy

Microwear data were acquired from the non-occlusal labial surface, as close to the apex as possible, of the mesial-most dentary tooth of dry skeletal specimens. The mesial-most tooth was chosen to ensure standardised sampling between unrelated reptiles as this tooth is straightforward to identify while determination of tooth homology between reptile taxa is problematic. Sampling this tooth also minimises possible confounding variation caused by differences in feeding behaviours (e.g. some crocodilians use their distally-positioned teeth to crush food items before swallowing^45^, whereas many lizards swallow items whole^87^). No preference was given to the left or right tooth and teeth from both sides were pooled in analyses. Teeth were cleaned using 70% ethanol-soaked cotton swabs to remove dirt and consolidant. High fidelity moulds were made using President Jet Regular Body polyvinylsiloxane (Coltène/Whaledent Ltd., Burgess Hill, West Sussex UK). This compound produces replica surfaces for textural analysis that are statistically indistinguishable from original tooth surfaces^88^. Initial moulds taken from each specimen were discarded to remove any remaining dirt with all analyses performed on second moulds. Casts were made from these moulds using EpoTek 320 LV Black epoxy resin, mixed to manufacturer’s instructions. Resin was cured for 24 hours under 200kPa (2 Bar/30 psi) of pressure (Protima Pressure Tank 10 L) to improve casting quality. Small casts were mounted onto 12.7 mm SEM stubs using President Jet polyvinylsiloxane with the labial, non-occluding surfaces orientated dorsally to optimise data acquisition. All casts were sputter coated with gold for three minutes (SC650, Bio-Rad, Hercules, CA, USA) to optimise capture of surface texture data.

### Surface texture data acquisition

Surface texture data acquisition follows standard laboratory protocols^22,33,68,88^. Data were captured using an Alicona Infinite Focus microscope G4b (IFM; Alicona GmbH, Graz, Austria; software version 2.1.2), using a x100 objective lens, producing a field of view of 146 × 100 micrometres. Lateral and vertical resolution were set at 440 nm and 20 nm respectively. Casts were orientated so labial surfaces were perpendicular to the axis of the objective lens.

All 3D data files were processed using Alicona IFM software (version 2.1.2) to remove dirt particles from tooth surfaces and anomalous data points (spikes) by manual deletion. Data were levelled (subtraction of least squares plane) to remove variation caused by differences in tooth surface orientation at the time of data capture. Files were exported as.sur files and imported into Surfstand (software version 5.0.0 Centre for Precision Technologies, University of Huddersfield, West Yorkshire, UK). Scale-limited surfaces were generated through application of a fifth-order robust polynomial to remove gross tooth form and a robust Gaussian filter (wavelength λ_c_ = 0.025 mm)^67,68^. International Organisation for Standardisation (ISO) 25178-2 areal texture parameters^35^ were then generated from each scale-limited surface. In-depth definitions and details of ISO parameters can be found in Supplementary Table S2.

### Statistical analyses

Log-transformed texture data were used for analyses as some of the texture parameters were non-normally distributed (Shapiro-Wilk, *P* > 0.05). The parameter Ssk was excluded from analyses as it contains negative values and thus could not be log-transformed.

To test the hypothesis that individuals from different dietary guilds exhibit different microwear textures, analysis of variance (ANOVA) with pairwise testing (Tukey HSD) was applied to each texture parameter.

Principal component analysis (PCA) was used to analyse texture parameters that exhibit significant differences between dietary guilds, creating a texture-dietary space. To test the hypothesis that microwear differences vary with dietary differences, we used Spearman’s rank to test for correlations between PC axes 1 and 2 and proportions in species diets of: total vertebrates, tetrapods, fish, total invertebrates, ‘harder’, ‘softer’ and ‘softest’ categories of invertebrates and plant matter. Total vertebrates (tetrapod and fish percentages summed) and total invertebrates were included in the dietary correlations to test the hypothesis that reptile tooth microwear records coarse dietary signals. PC axes 1 and 2 were also each correlated against dietary generalism, i.e. numbers of different food items in reptile diets. The material properties of invertebrate exoskeletons are better quantified than vertebrate integuments^59–62^, thus vertebrate food items were assigned to dietary groups at Class level and invertebrate prey were assigned at Order level. Plant material included leaves, fruits and seeds.

Additional analyses were employed to test the relationships between texture and diet in the texture-dietary space and to further test for textural differences between guilds. We used Spearman’s rank to test for correlations between parameter values of all sampled teeth and PCs 1 and 2, for each parameter. Average values of each parameter were calculated for each guild, and these were separately ranked between guilds, from most to least positive (see Supplementary Table S6 for all rankings). Matched pairs t-tests were used to compare the profiles of average parameter values between guilds.

A Benjamini-Hochberg (B-H) procedure was used to account for the possibility of inflated Type I error rates associated with multiple comparisons^89^. The False Discovery rate was set at 0.05. The B-H procedure was not needed for the Tukey HSD tests as it already accounts for inflated Type I error rates^27^.

All analyses were performed with JMP Pro 12 (SAS Institute, Cary, NC, USA) except for the Benjamini-Hochberg procedure, which used Microsoft Excel^90^; www.biostathandbook.com/multiplecomparisons.html).

## Supplementary information

Supplementary Info

Table S1.

Table S7.

## Data Availability

The datasets generated from the current study are available from the corresponding authors upon request.
