# Supplementary material for: Dietary differences in archosaur and lepidosaur reptiles revealed by dental microwear textural analysis
Source: Sci Rep. 2019 Aug 12;9:11691. doi: 10.1038/s41598-019-48154-9 (PMC6690991; doi:10.1038/s41598-019-48154-9)
Supplement: Supplementary file 1 — Supplementary Info [file 41598_2019_48154_MOESM1_ESM.pdf]

**Supplementary Information for: Dietary differences in archosaur and lepidosaur reptiles revealed by dental microwear textural analysis.**

Jordan Bestwick[1]*, David M. Unwin[2] and Mark A. Purnell[1]*

[1]School of Geography, Geology and the Environment, University of Leicester, Leicester, LE1 7RH, United Kingdom.

[2]School of Museum Studies, University of Leicester, Leicester, LE1 7RF, United Kingdom.

*Corresponding author emails: jb656@leicester.ac.uk; map2@leicester.ac.uk

**Supplementary Text**

**Tukey HSD pairwise test results of the significant ISO parameters between reptile dietary guilds**

ANOVA tests of the roughness parameters reveal that four parameters significantly differed between reptile dietary guilds: Spk, Sds, Vmp and Smr1 (Table 2). Tukey HSD pairwise testing of the significant roughness parameters indicates that carnivores significantly differ from ‘harder’ invertebrate consumers for one parameter (higher Sds), but do not differ from ‘softer’ invertebrate consumers, omnivores or piscivores. ‘Harder’ invertebrate consumers differ from piscivores for three parameters (higher Vmp, Spk and Smr1); differ from ‘softer’ invertebrate consumers for one parameter (lower Sds); but do not differ from omnivores. ‘Softer’ invertebrate consumers do not differ from omnivores or piscivores. Omnivores differ from piscivores for three parameters (higher Vmp, Spk and Smr1).

**Table S2. International Organisation for Standardisation (ISO) 3D texture parameter definitions and categorizations.** Many parameters are derived from the areal material ratio curve; a cumulative probability density function derived from the scale-limited tooth surface by plotting the cumulative percentage of the tooth surface against height. Figure S2 provides a graphical explanation. The peaks, valleys and core material of tooth surfaces are defined on the basis of this curve, with the core for material ratio parameters equivalent to the volume that lies between the heights of the surface delimited by the extrapolated intercept of the minimum slope of the curve (see Figure S2). 'Core', peaks and valleys for volume parameters are defined using slightly different thresholds.

| para-meter | unit | definition | | |
|---|---|---|---|---|
| Sq | µm | Root-Mean-Square height of surface | height | Sq, root mean square of height, provides an overall measure of the height of the texture comprising the surface |
| Sp | µm | Maximum peak height of surface | height | Sp, the height of the highest peak, tends not to be reliable as an indicator of overall surface texture, as it is based on only one peak, which could reflect a single point. |
| Sv | µm | Maximum valley depth of surface | height | Sv, the depth of the deepest valley or pit, tends not to be reliable as an indicator of overall surface texture, as it is based on only one valley or pit, which could reflect a single point. |
| Sz | µm | Maximum height of surface | height | Sz, the maximum height of the surface, is calculated by subtracting the maximum valley depth from the peak height |
| Sa | µm | Average height of surface | height | Sa, the average height of the surface, provides an overall measure of the height of the texture comprising the surface |
| Ssk | - | Skewness of height distribution of surface | height | Ssk is the skewness of the 3D surface texture, measuring the deviation from normality of the distribution of heights of all points in a measured area. Ssk captures the degree of symmetry of the surface heights about the mean plane, with positive values indicating a predominance of peaks and negative values indicating a predominance of valleys. |
| Sku | - | Kurtosis of height distribution of surface | height | Sku is the kurtosis of the 3D surface texture, measuring the deviation from normality of the distribution of heights of all points in a measured area. A surface with a normal distribution of height will have a Sku value of 3; a spikey surface, with inordinately high peaks or deep valleys, will have a value of less than 3. |
| S5z | µm | 10 point height of surface | feature | S5z is the average value of the 5 highest peaks and the 5 deepest valleys, providing a measure of extremes of height |
| Sdq | - | Root mean square gradient of the surface | hybrid | Higher values of Sdq indicate steeper gradients of the slopes comprising the surface. It is affected by both the amplitude and spacing of texture, with more closely spaced texture generally having steeper slopes. |
| Sdr | % | Developed interfacial area ratio | hybrid | Sdr is the % additional contribution of the texture to surface area, relative to a plane of the same linear dimensions. Higher values for Sdr indicate more intricate, more complex textures. |
| Sds | $1/mm^2$ | Density of summits. Number of summits per unit area making up the surface | hybrid | Increases in Sds indicate that peaks above the core material make up a greater proportion of the surface |
| Ssc | 1/µm | Mean summit curvature for peak structures | | Increases in Ssc indicate that peaks above the core material have more rounded summits |
| Sk | µm | Core roughness depth, Height of the core material | material ratio | The vertical distance between the low and high limits of the core material (defined as outlined in table caption and Figure S2) |
| Spk | µm | Mean height of the peaks above the core material | material ratio | Spk is the mean height of peaks above the top of the core, with high values indicating a surface composed of high peaks |
| Svk | µm | Mean depth of the valleys below the core material | material ratio | Spk is the mean depth of valleys below the base of the core, with high values indicating a surface composed of deep valleys |
| Smr1 | % | Surface bearing area ratio (the proportion of the surface which consists of peaks above the core material) | material ratio | The percentage of the surface that is composed of the peaks that are higher than the top of the core. |
| Smr2 | % | Surface bearing area ratio (the proportion of the surface which would carry the load) | material ratio | The percentage of the surface that is composed of the valleys that are lower than the base of the core. |
| Vmp | $\mu m^3/mm^2$ | Material volume of the peaks of the surface | volume | The volume of material contained within peaks that make up the highest 10% of the surface |
| Vmc | $\mu m^3/mm^2$ | Material volume of the core of the surface | volume | The volume of the material making up the surface, excluding peaks (the highest 10%) and valleys (lowest 20% of the surface). 'Core' in the context of volume parameters is not defined in the same way as core for material ratio parameters. |
| Vvc | $\mu m^3/mm^2$ | Void volume of the core of the surface | volume | The volume of the voids within the 'core' of the surface, the core excluding peaks (the highest 10%) and valleys (lowest 20% of the surface). |
| Vvv | $\mu m^3/mm^2$ | Void volume of the valleys of the surface | volume | The volume of voids contained within valleys that make up the lowest 20% of the surface. |
| Sal | mm | Auto correlation length. | spatial | Horizontal distance of the auto correlation function (ACF) which has the fastest decay to the value 0.2. Large values for Sal indicate surfaces dominated by low frequencies. Small values indicate surfaces dominated by high frequencies |
| Str | - | Texture aspect ratio (values range 0-1). | spatial | The ratio from the distance with the fastest decay of the auto correlation function to the distance with the slowest decay. Values of 0.2-0.3 indicate surfaces with a strong directional structure. Values of > 0.5 indicate a rather uniform texture. |

**Table S3. Reptile dietary correlations.** Spearman rank correlations of PC axes 1 and 2 against dietary characteristics. Significant correlations after application of the Benjamini-Hochberg procedure shown in bold.

| **Correlation** | $r_s$ | ***P*-value** | **d.f.** |
|---|---|---|---|
| **PC1 – Total vertebrates** | **-0.3564** | **0.0004** | **94** |
| PC2 – Total vertebrates | 0.0769 | 0.459 | 94 |
| PC1 – Tetrapods | -0.063 | 0.5439 | 94 |
| PC2 – Tetrapods | 0.2072 | 0.0439 | 94 |
| PC1 – Fish | -0.2398 | 0.0192 | 94 |
| PC2 – Fish | -0.1723 | 0.0949 | 94 |
| **PC1 – Total invertebrates** | **0.3192** | **0.0016** | **94** |
| PC2 – Total invertebrates | 0.0769 | 0.459 | 94 |
| PC1 – ‘Harder’ invertebrates | 0.25 | 0.0145 | 94 |
| PC2 – ‘Harder’ invertebrates | 0.0348 | 0.7381 | 94 |
| PC1 – ‘Softer’ Invertebrates | 0.1786 | 0.0833 | 94 |
| **PC2 – ‘Softer’ invertebrates** | **0.2907** | **0.0043** | **94** |
| PC1 – ‘Softest’ invertebrates | 0.08 | 0.4409 | 94 |
| PC2 – ‘Softest’ invertebrates | 0.0377 | 0.7165 | 94 |
| PC1 – Plant matter | 0.0671 | 0.518 | 94 |
| PC2 – Plant matter | -0.0616 | 0.5533 | 94 |
| PC1 – Generalism | 0.0755 | 0.4672 | 94 |
| PC2 – Generalism | 0.145 | 0.1609 | 94 |

**Table S4. Reptile textural correlations.** Spearman rank correlations of texture parameter values for reptile dietary guilds against PC axes 1 and 2 derived from the four texture parameters that differ between reptile guilds. Significant correlations after application of the Benjamini-Hochberg procedure shown in bold.

| **Parameter** | **PC 1 $r_s$** | ***P*-value** | **PC 2 $r_s$** | ***P*-value** |
|---|---|---|---|---|
| Sq | **0.6798** | **<0.0001** | -0.1865 | 0.0704 |
| Sku | **0.2762** | **0.0067** | 0.0089 | 0.9315 |
| Sp | **0.7560** | **<0.0001** | 0.1448 | 0.1614 |
| Sv | **0.326** | **0.0013** | -0.0217 | 0.8349 |
| Sz | **0.6646** | **<0.0001** | 0.0494 | 0.6345 |
| Sds | -0.0249 | 0.8108 | **0.986** | **<0.0001** |
| Str | -0.1877 | 0.1512 | **0.2353** | **0.0217** |
| Sdq | **0.4004** | **<0.0001** | **0.2945** | **0.0038** |
| Ssc | **0.4289** | **<0.0001** | **0.5378** | **<0.0001** |
| Sdr | **0.4019** | **<0.0001** | **0.2899** | **0.0044** |
| Vmp | **0.9763** | **<0.0001** | 0.0596 | 0.5662 |
| Vmc | **0.5798** | **<0.0001** | -0.138 | 0.1825 |
| Vvc | **0.7864** | **<0.0001** | -0.0152 | 0.8835 |
| Vvv | 0.1876 | 0.0687 | **-0.31** | **0.0022** |
| Spk | **0.969** | **<0.0001** | 0.0634 | 0.5416 |
| Sk | **0.6066** | **<0.0001** | -0.0682 | 0.5115 |
| Svk | 0.2005 | 0.0514 | **-0.2899** | **0.0044** |
| Smr1 | **0.8024** | **<0.0001** | **0.2681** | **0.0086** |
| Smr2 | **0.5273** | **<0.0001** | **0.3199** | **0.0016** |
| S5z | **0.631** | **<0.0001** | 0.0015 | 0.9883 |
| Sa | **0.6662** | **<0.0001** | -0.1729 | 0.0939 |

**Table S5. Matched-pairs t-test results (4 d.p) of the means of ISO texture parameters between dietary guilds.** Data log transformed and scale limited using 5th order polynomial and robust Gaussian filter. Pairwise tests exhibiting significant differences after application of the Benjamini-Hochberg procedure shown in bold. Abbreviations: C, Carnivores; HI; 'Harder' Invertebrate consumers, O; Omnivores, P; Piscivores, SrI; 'Softer' Invertebrate consumers.

| **Pairwise test** | ***t*-ratio** | ***P*-value** | **d.f** |
|---|---|---|---|
| **C-HI** | **2.9474** | **0.008** | **20** |
| C-O | 1.3494 | 0.1923 | 20 |
| **C-P** | **-3.6765** | **0.0015** | **20** |
| C-SrI | 0.2162 | 0.831 | 20 |
| HI-O | -0.7425 | 0.4664 | 20 |
| **HI-P** | **-4.874** | **<0.0001** | **20** |
| HI-SrI | -2.0831 | 0.0503 | 20 |
| **O-P** | **-2.6508** | **0.0153** | **20** |
| O-SrI | 1.0559 | 0.3036 | 20 |
| **P-SrI** | **-3.4747** | **0.0024** | **20** |

**Table S6. ISO texture parameter average value rankings between reptile dietary guilds.** 1 = most positive parameter values, 5 = least positive values. Parameters in bold are used in the principal component analysis (Fig. 2). Average rank of all parameters per guild to 4 d.p. Abbreviations: C, Carnivores; HI; 'Harder' Invertebrate consumers, O; Omnivores, P; Piscivores, SrI; 'Softer' Invertebrate consumers. For ISO parameter definitions, see Supplementary Table S3.

| Parameter | C | HI | O | P | SrI |
|---|---|---|---|---|---|
| Sq | 3 | 2 | 1 | 5 | 4 |
| Sku | 4 | 2 | 1 | 3 | 5 |
| Sp | 3 | 2 | 1 | 5 | 4 |
| Sv | 5 | 2 | 1 | 4 | 3 |
| Sz | 3 | 2 | 1 | 5 | 4 |
| **Sds** | **2** | **5** | **3** | **4** | **1** |
| Str | 3 | 4 | 5 | 2 | 1 |
| Sdq | 2 | 3 | 5 | 4 | 1 |
| Ssc | 2 | 3 | 5 | 4 | 1 |
| Sdr | 2 | 3 | 5 | 4 | 1 |
| **Vmp** | **3** | **2** | **1** | **5** | **4** |
| Vmc | 3 | 1 | 5 | 4 | 2 |
| Vvc | 4 | 1 | 2 | 5 | 3 |
| Vvv | 4 | 1 | 3 | 2 | 5 |
| **Spk** | **3** | **2** | **1** | **5** | **4** |
| Sk | 3 | 1 | 5 | 4 | 2 |
| Svk | 4 | 1 | 2 | 3 | 5 |
| **Smr1** | **3** | **2** | **1** | **5** | **4** |
| Smr2 | 2 | 4 | 1 | 5 | 3 |
| S5z | 3 | 1 | 2 | 5 | 4 |
| Sa | 3 | 1 | 2 | 5 | 4 |
| Average Rank | 3.0476 | 2.1429 | 2.5238 | 4.1905 | 3.0952 |

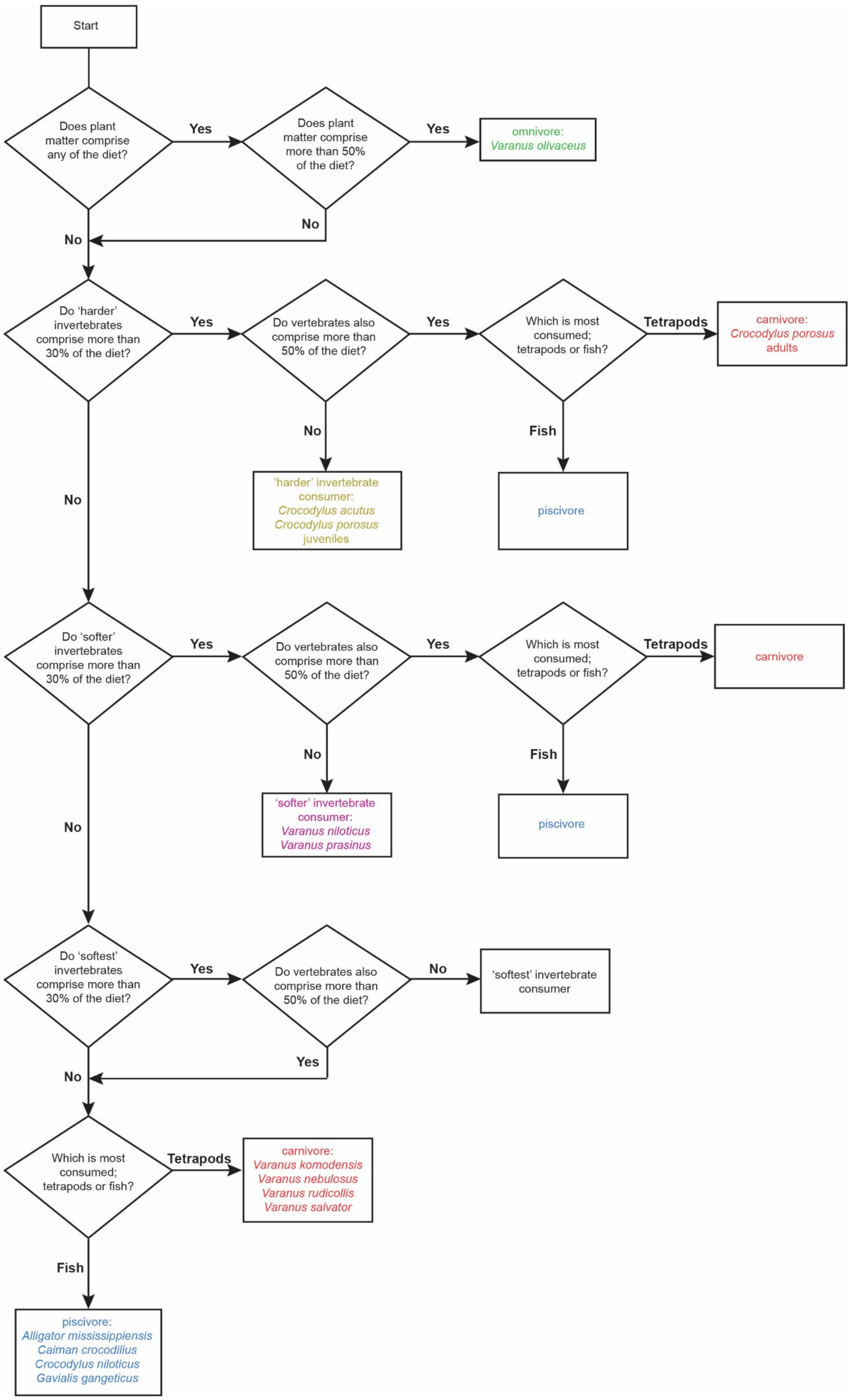
Start
Does plant matter comprise any of the diet?
Yes
Does plant matter comprise more than 50% of the diet?
Yes
omnivore: *Varanus olivaceus*
No
No
Do 'harder' invertebrates comprise more than 30% of the diet?
Yes
Do vertebrates also comprise more than 50% of the diet?
Yes
Which is most consumed; tetrapods or fish?
Tetrapods
carnivore: *Crocodylus porosus* adults
No
Fish
'harder' invertebrate consumer: *Crocodylus acutus* *Crocodylus porosus* juveniles
piscivore
No
Do 'softer' invertebrates comprise more than 30% of the diet?
Yes
Do vertebrates also comprise more than 50% of the diet?
Yes
Which is most consumed; tetrapods or fish?
Tetrapods
carnivore
No
Fish
'softer' invertebrate consumer: *Varanus niloticus* *Varanus prasinus*
piscivore
No
Do 'softest' invertebrates comprise more than 30% of the diet?
Yes
Do vertebrates also comprise more than 50% of the diet?
No
'softest' invertebrate consumer
Yes
No
Which is most consumed; tetrapods or fish?
Tetrapods
carnivore: *Varanus komodensis* *Varanus nebulosus* *Varanus rudicollis* *Varanus salvator*
Fish
piscivore: *Alligator mississippiensis* *Caiman crocodilius* *Crocodylus niloticus* *Gavialis gangeticus*

**[From previous page] Fig. S1. Schematic overview of how study reptiles were classified into dietary guilds.** Classification system adapted from refs.[6,9,11,29,73–76]. Reptile dietary information derived from stomach and/or faecal content studies which provide dietary constituents as volumetric or frequency proportions of total diet. *Crocodylus porosus* adults, as termed in this study, are specimens whose total length exceeds 3.5 m[85]. Specimens with total lengths below 3.5 m are termed juveniles in this study[85]. Invertebrates are defined as soft, intermediate or hard based on the relative difficulty for teeth to pierce the exoskeleton, as determined by experimental work from refs.[59–62]. See Methods for a full description of which invertebrates were categorised as 'harder', 'softer' and 'softest'. For full dietary breakdowns and sources of dietary information, see Supplementary Table S1.

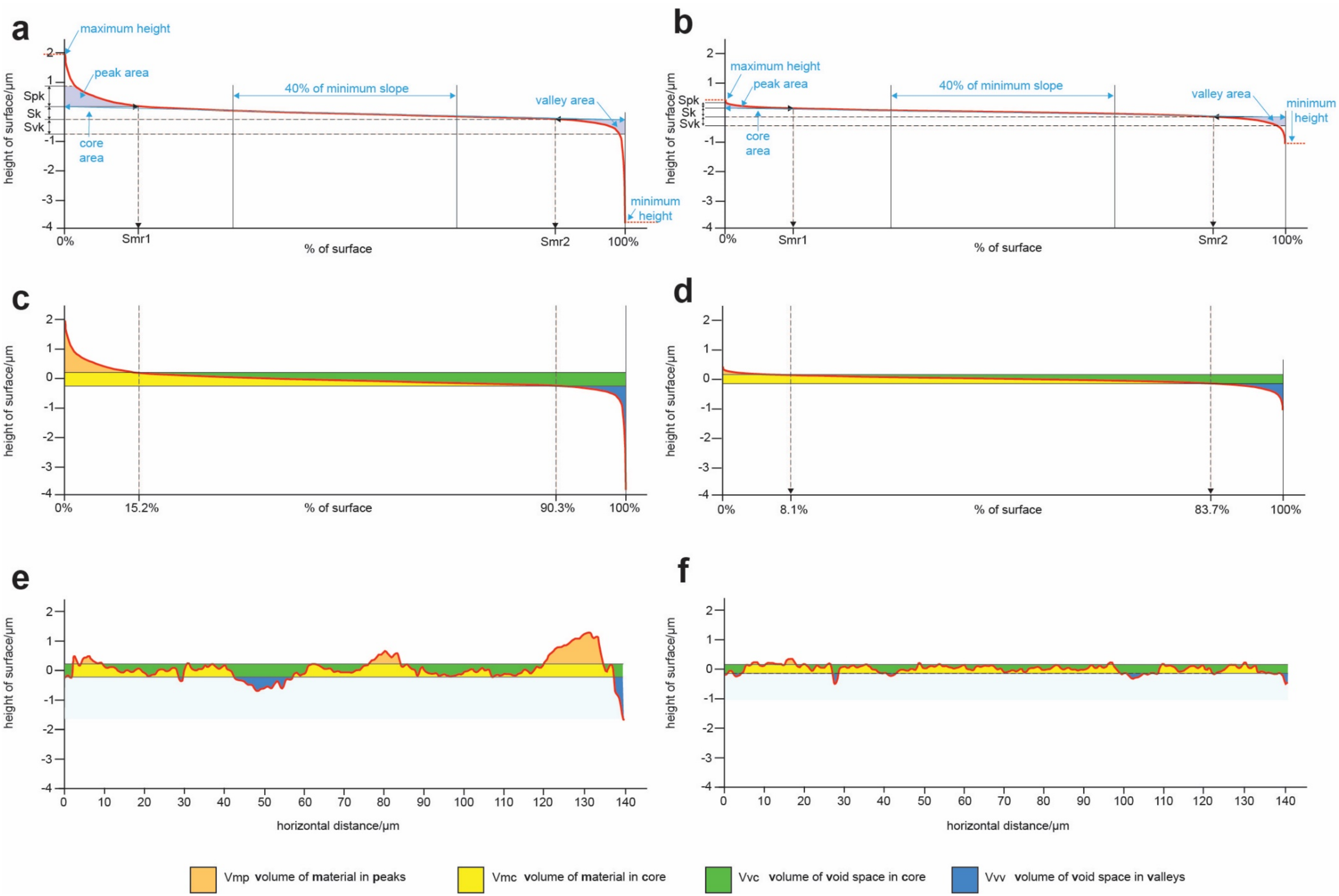
a
maximum height
peak area
40% of minimum slope
valley area
Spk
Sk
Svk
core area
minimum height
Smr1
Smr2
0%
100%
% of surface
height of surface/µm
b
c
15.2%
90.3%
d
8.1%
83.7%
e
f
horizontal distance/µm
Vmp volume of material in peaks
Vmc volume of material in core
Vvc volume of void space in core
Vvv volume of void space in valleys

**[From previous page] Fig. S2. Graphical representation of a number of ISO areal texture parameters in an omnivore (*Varanus olivaceus*; IFM No. 214100) and a piscivore (*Gavialis gangeticus*; IFM No. 123600) tooth surface. a–d,** The Areal Material Ratio Curve, from which a number of height, volume and material ratio parameters are derived, in *V. olivaceus* (a, c) and *G. gangeticus* (b, d). For full parameter definitions see Supplementary Table S3. The curve is a cumulative probability density function, derived from the scale limited surface by plotting the cumulative percentage of the surface against height. Core, peaks and valleys within a surface are defined on the basis of this curve, with the core equivalent to the volume that lies between the heights of the surface delimited by the extrapolated intercept of the minimum slope of the curve as shown in the figure. **e, f,** Schematic cross section through the omnivore and piscivore tooth surfaces, respectively, showing how volume parameters relate to a surface. All panels to the same scale. The material ratio curve is applied to the whole tooth surface, hence the maximum and minimum surface heights in panels a–d are different to panels e–f which are cross-sections of a single plane across the tooth surface. Note that the schematics are presented in 2D but volume parameters are calculated for the whole 3D surface.
